# The impact of microplastics on the mice gut microbiome: a meta-analysis

**DOI:** 10.1038/s41598-026-49190-y

**Published:** 2026-04-22

**Authors:** Anika Kalra, Davide Dominoni, Jelle Boonekamp

**Affiliations:** School of Biodiversity, One Health & Veterinary Medicine, Graham Kerr Building, University of Glasgow, Glasgow, UK

**Keywords:** Microplastics, Nanoplastics, Gut microbiome, Shannon index, Relative abundance, Ecology, Ecology, Environmental sciences, Microbiology

## Abstract

Microplastics are nearly ubiquitous in the natural environment. There is increasing awareness of the ingestion of microplastics in a wide range of wild animals and humans, but their health and fitness impacts remain poorly understood. Mounting evidence suggests that microplastics could accumulate in the gut, affecting the microbiome and hindering its function. This may constitute a direct pathway through which exposure to microplastics could affect health and disease. However, prior studies on the effects of microplastic exposure on the microbiome show contrasting findings raising the question whether microplastics truly affect the microbiome. Here, we performed the first meta-analysis on the effects of dietary microplastic exposure on the gut microbiome of mice. Overall, we found support for a significant impact of microplastic exposure on the relative abundance of gut bacteria. Microplastic exposure significantly increased the relative abundance of phyla Bacillota and Pseudomonadota. More in depth order-level analysis revealed significant increases in relative abundance within Bacillota of orders Lachnospirales and Lactobacillales and within Pseudomonadota of orders Burkholderiales, Hyphomicrobiales and Moraxellales. In the gut microbiome, Bacillota is associated with carbohydrate metabolism and Pseudomonadota is associated with facultative anaerobes, thus these changes may have functional consequences. Our findings highlight the importance of distinguishing among differing bacterial taxa when assessing the impacts of microplastics on the gut microbiome as the direction and size of the impact varies by phyla.

**Supplementary Information:**

The online version contains supplementary material available at 10.1038/s41598-026-49190-y.

## Background

Mass produced plastics have gradually accumulated since the 1950’s and are now considered globally ubiquitous in the natural environment^1–8^. Plastic products break down from natural causes and form “microplastics”, defined by most literature as less than 5 mm, which subsequently break down into “nanoplastics” classed as less than 1 µm^3,9,10^. Micro- and nano-plastic particles (henceforth called microplastics) have been found in human food sources such as alcohol, coffee, sugar, honey, salt, bottled water, and tap water, as well as chicken and sheep faeces^6,11–16^. Microplastics have also been widely reported in marine environments and have been detected in the soft tissue of mussels and oysters^16^. There is growing concern for the health^17^ and reproductive^17,18^ implications of plastic ingestion in wild animals, livestock, and humans, as the negative effects of exposure to microplastics are becoming increasingly apparent, but the underlying mechanisms remain unclear.

The main route of microplastics exposure for mammals and humans occurs through ingestion, resulting in the accumulation of microscopic plastic particles in the gastrointestinal tract^4,6^. Microplastics have been detected in human stool, suggesting the partial clearance of microplastic particles through faecal excretion^19^. Nevertheless, it has been hypothesized that the presence of microplastics in the gut causes negative health impacts through their effects on the gut microbiome composition underpinning chronic disease, metabolic dysfunction, oxidative stress, and inflammation^3–7,10,20,21^. Previous experimental studies on the toxicological impacts of microplastics on gut microbiome parameters have almost exclusively focused on laboratory mice and rat systems^16,22^. Several of these studies support that experimental microplastics exposure, for example through the diet, results in the accumulation of microplastics in the gut^6^, liver and kidney^23^, and can induce intestinal microbial dysbiosis through changes in the relative abundance of gut microbiome bacteria and species diversity^5,23–25^. However, other studies reported no effects of microplastics exposure on the microbiome^22,26^ or different types of changes indicative of dysbiosis^6^. Han et al.^26^ suggests the lack of significant biological and histopathological effects of microplastics exposure found may have been due to experimental differences in dosage and exposure duration. We conclude therefore that it remains unclear whether microplastics affect the gut microbiome and if such effects depend on the dosage and particle size of the microplastics that were used.

Here, we conduct the first meta-analysis on the impacts of microplastics’ exposure on the mice gut microbiome, and whether such effects depend on dosage and particle size. We confined our meta-analysis to experimental laboratory mice studies as they are by far the most common model of studying the toxicological impact of microplastics and are the main model for translation to human diseases^16,22^. We hypothesize that if microplastics exposure would disrupt the gut microbiome dynamics through changes in the relative abundance of gut microbiome bacteria and species diversity, then this should lead to an interspecific competition imbalance^8,27,28^. If so, then certain bacteria will be outcompeted whilst others thrive, resulting in a reduced microbiome diversity. We also hypothesize increased relative abundance of competitive gut microbiome bacteria and we anticipate these effects to be exacerbated by higher plastic dosages as well as smaller plastic sizes, because smaller particles might penetrate more easily into the epidermal tissues^23,29^.

## Methods

### Data collection

**Fig. 1:**
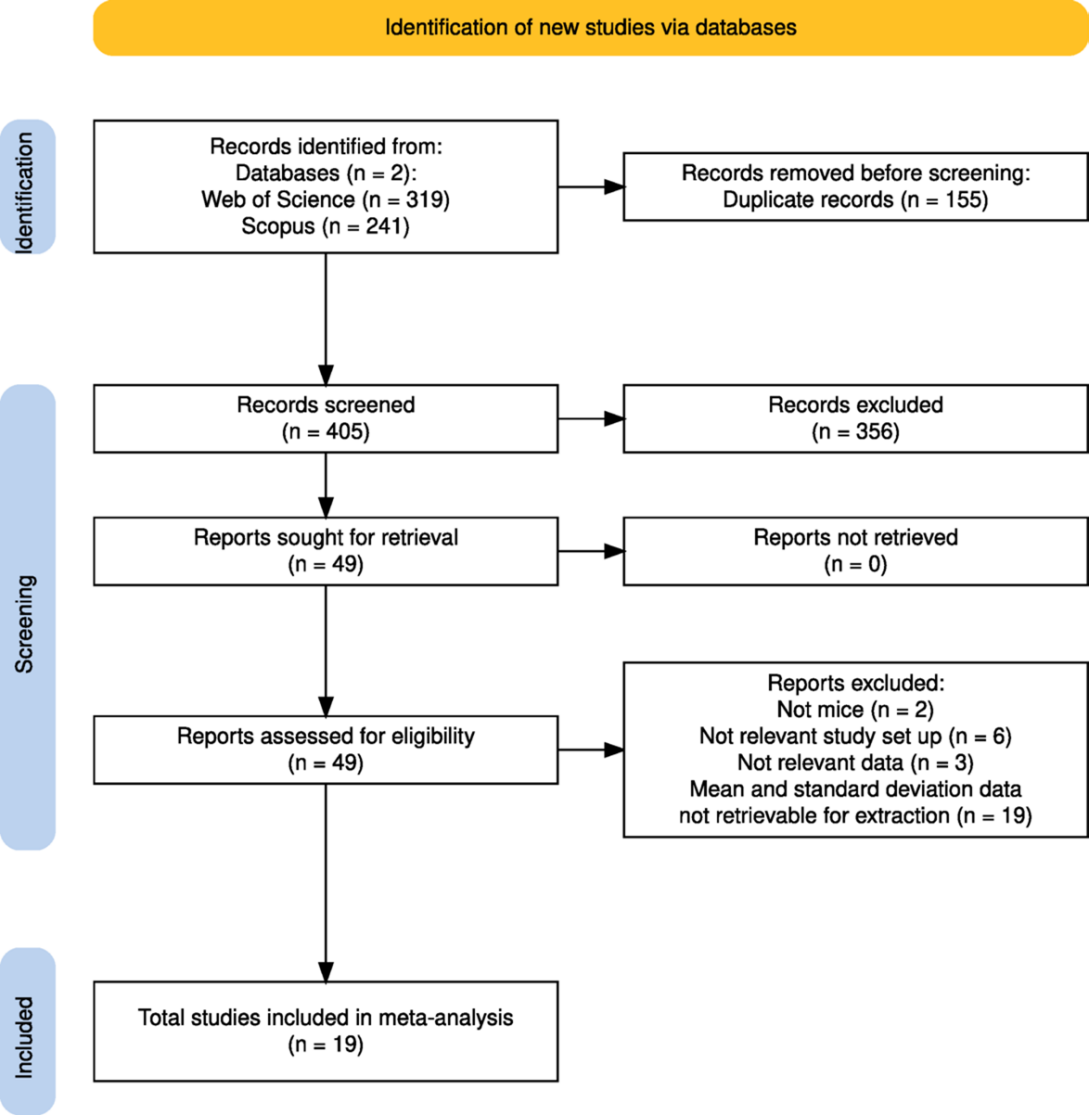
Preferred Reporting Items for Systematic Reviews and Meta-Analyses (PRISMA) diagram of screened studies for meta-analysis. Adapted from^31^.

We performed a literature search on the Web of Science and Scopus ending on May 4^t+h^, 2024 and May 16th, 2024, respectively. Our original aim was to capture all research papers on the effects of experimental microplastic exposure on the gut microbiome in terrestrial vertebrates, but during the screening of the literature, it became apparent that the vast majority of suitable papers were on laboratory mice, likely because these are highly suitable systems amenable to experimental exposure to microplastics. Hence, in chronological order, our literature search was conducted as follows: we used the search strings: “microplastics NOT fish NOT invertebrate NOT (mollusc OR arthropod OR worm OR cnidarian OR echinoderm OR sponge) NOT marine NOT aquatic NOT soil AND (microbiome OR microbe)” and “microplastics AND (bird OR mammal OR reptile OR amphibian) AND (microbiome OR microbe)”. This resulted in a total of 405 potentially suitable papers after duplicates across Web of Science and Scopus were removed (Fig. 1). Subsequently, we screened the titles and abstracts for relevancy followed by an in-depth screening of the relevant papers. We adopted the PECO (Population/Exposure/Comparison/Outcomes) format as a framework for applying the inclusion criteria^30^. The exclusion criteria included non-terrestrial species, aquatic, marine or soil environments, engineered treatments or patents, “plasticity” of gut microbiome, introduction of any other independent variables besides microplastic exposure, and microplastic exposure impact on variables outside of the gut microbiome. The first round of screening resulted in 94 papers. At this point, the research question was refined to focus only on mice, resulting in 49 papers (Fig. 1). Out of the 45 papers that were removed in this refinement, only 3 papers (2 studies on chicken and 1 study on toads) would have been suitable considering the original inclusion and exclusion criteria. For the second round of screening, we read the full paper in detail to confirm relevancy and suitability regarding the published data and study design defined as experimental mice studies where there was at least one control group, one plastic particle only treatment group, and microbiome analysis. The second screening round resulted in 38 papers. Finally, if mean and standard deviation data for relative abundance per gut bacteria phyla and/or Shannon’s index was not retrievable, those papers were excluded, resulting in 19 papers ultimately included in our meta-analysis (Fig. 1). See Supplementary Table 1, Additional File 1 for the extracted data by paper ultimately used in our meta-analysis (Table S1).

Gut microbiome dynamics are often expressed in terms of relative abundance for each taxon included in the study, and taxa alpha diversity indices such as Shannon’s index (Kers and Saccenti 2021). Therefore, we split our meta-analysis into two separate analyses for each metric as they provide complementary information about the impacts of plastics on the microbiome, though not all papers provided information on both metrics. We obtained effect sizes by extracting the means and standard deviations for Shannon’s index and relative abundance per taxon from each paper as follows: we either used the published sample sizes, means, and standard deviations (or 95% confidence intervals from which SDs were derived), or we contacted the authors in case these statistics were missing. If authors did not respond, we used WebPlotDigitizer 5.0^32^ to extract the means and standard deviations from the data figures. In this case, we were limited to using bar plots showing the means and standard deviations, e.g. boxplots were excluded. This meant the final number of effect sizes included was n = 246 from 13 studies for relative abundance, and n = 17 effect sizes from 11 studies for Shannon’s Index, (Fig. 1). There were many more relative abundance effect sizes than Shannon’s Indices because relative abundance was typically estimated for each phylum or species, etc., and each study included relative abundance estimates for multiple phyla or species, depending on the selected taxonomic class of the study. There were, at times, multiple Shannon’s Index effect sizes for a particular study if a study had multiple treatment groups with varying plastic sizes or daily dosages.

We also extracted data on plastic particle size, daily dosage of the exposure, and duration of time of the exposure, if this information was provided, to test their effects as moderators in our meta-analyses. We were limited in publicly available data to extract rarefaction transformation to sequencing data depth. For the relative abundance data, if there were multiple treatment groups of different dosages, we included the highest dosage treatment group. If the dosages were equivalent and there were multiple experimental groups of different plastic sizes, we used the smallest plastic size treatment group. If there were multiple bacterial taxa ranks analysed, we used the most specific taxa rank (i.e. if genus and species were analysed, we used species) and then re-labelled taxa at the phyla and order level where possible (i.e., if only analysed as phyla level, we may not have had the specificity to re-label to order level) to enable including phyla and order as moderator variables in our meta-analysis. For plastic size, we took the average value (μm) if a range of plastic sizes were used. For treatment dosage, we used the mg plastics per day scaled to body mass^33^. If body weight was not published, we extracted the average body weight from body weight figures using WebPlotDigitizer for each treatment group. All extracted data, whether used in this meta-analysis or not, can be found in the supplementary information.

We used the escalc function in the metafor package (v4.8.0)^34^ to calculate the standardised mean differences between the control and treatment groups expressed as Hedges’ g^35^:

$$Hedge{s}{\prime}g= \frac{m1-m2 }{\sqrt{\frac{\left(\left(n1-1\right)*{sd1}^{2}\right)+((n2-1)*{sd2}^{2})}{(n1+n2-2)}}}$$

where *m* refers to the mean, *n* refers to the sample size, *sd* refers to the standard deviation, and the numerical labelling refers to the treatment group (1) and the control group (2), i.e. positive effect sizes indicate that microplastic treatment increased gut bacteria metrics.

### Meta-analysis

We used metafor (v4.8.0)^34^ in R (v4.5.2) to run multivariate models including study ID and observation ID as random effects^36^. We subsequently added the moderators of plastic size, treatment dosage, and phyla to estimate their respective effects. We used Q-tests to evaluate the heterogeneity among study effect sizes. We tested two different forms of publication biases: bias against publishing small sample size studies without significant results and timing bias against publishing statistically non-significant results. All figures were made using the orchaRd package (v2.1.3)^36^ and select figures modified with Inkscape^37^. See Additional files 2–5 for raw data and meta-analysis R scripts.

## Results

### Effect of microplastics on gut bacteria species alpha diversity

**Fig. 2:**
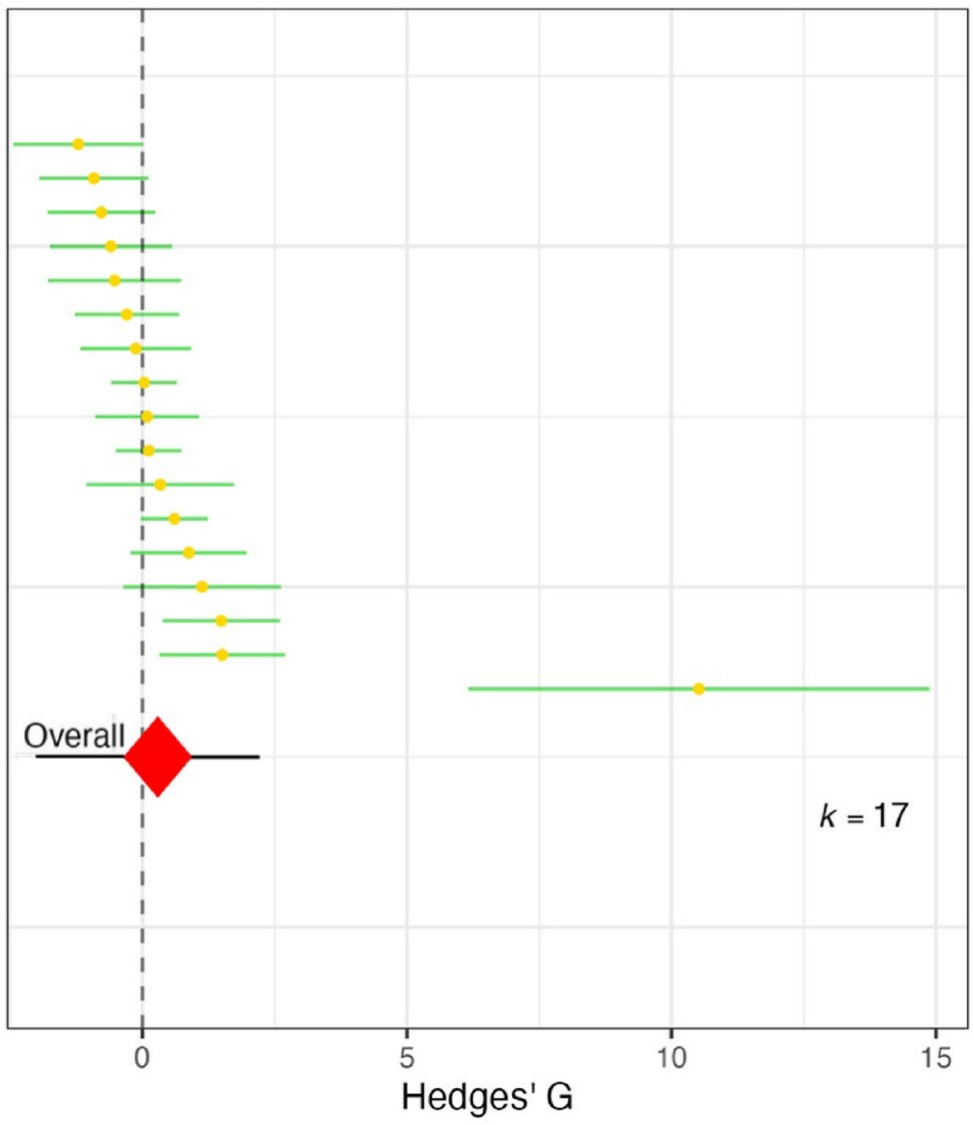
Forest plot of Hedges’ g effect sizes of microplastics impact on gut bacteria alpha diversity. Yellow circles show the meta-analysis effect size point estimate for each set of control vs. treatment means, i.e. positive effect sizes indicate microplastic treatment increased gut bacteria alpha diversity (Shannon index). Horizontal bars indicate the predictability interval. The red diamond indicates the overall effect size estimate where diamond width reflects the 95% confidence interval. *K* denotes the total effect size count.

**Fig. 3:**
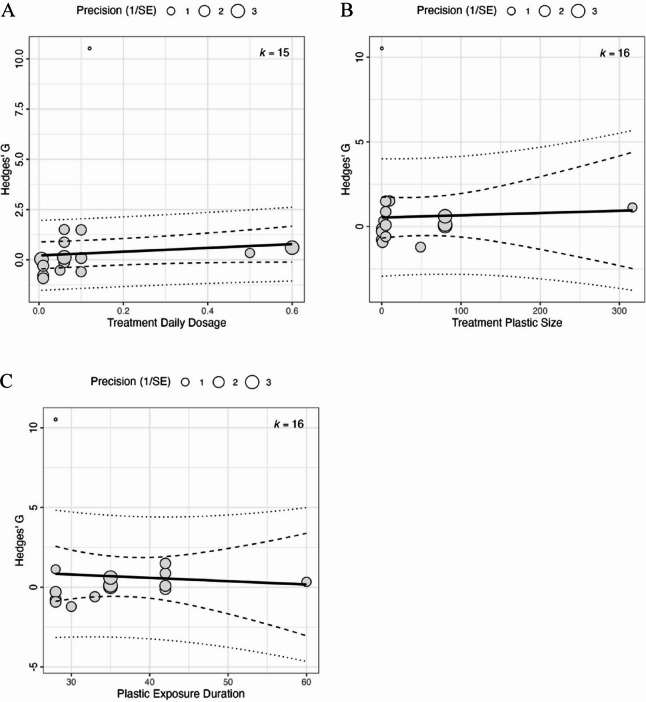
Moderator estimates in the meta-analysis of microplastics impact on gut bacteria species alpha diversity. (**A**) Effect of plastic particles daily dosage on gut bacteria species alpha diversity. (**B**) Effect of particle size on gut bacteria species alpha diversity. (**C**) Effect of plastic exposure duration on gut bacteria species alpha diversity. The bold lines indicate the moderators’ regression lines. The dashed lines indicate the 95% CI and the dotted lines indicate the predictability intervals. *K* denotes the total number of effect sizes in the moderator analyses. Bubble size reflects the weight of the effect size in the meta-analysis. Gut bacteria alpha diversity is estimated by Shannon diversity index.

Dietary microplastics did not significantly affect gut bacteria species alpha diversity (0.289, 95% CI − 0.362 to 0.941, z-value = 0.871, *p* = 0.384, Fig. 2). There was significant heterogeneity (Q = 53.728, *p* < 0.001), indicating substantial variation among effect sizes. We therefore investigated if our moderators, treatment plastic daily dosage, treatment plastic particles size, and treatment plastic exposure duration could explain some of this variation. However, our moderator analysis revealed the daily dosage of plastic particles in the treatment group was not significant (0.941, 95% CI − 0.364 to 2.247, z-value = 1.413, *p* = 0.158, Fig. 3A), the size of plastic particles was not significant (0.001, 95% CI − 0.011 to 0.013, z-value = 0.216, *p* = 0.829, Fig. 3B), and nor was the duration of plastic exposure (-0.021, 95% CI − 0.151 to 0.109, z-value = − 0.316, *p* = 0.752, Fig. 3C). We found adding these moderators reduced heterogeneity compared to the intercept only model, but the residual heterogeneity remained substantial (QE = 589.801, *p* < 0.001). One study showed a markedly larger effect size than the rest. Upon closer inspection, the treatment effect appeared similar when comparing the means across treatment groups, however the standard deviations were much smaller compared with the other studies, resulting in a larger Hedges’ g effect size value. We could not identify a specific reason for the much smaller standard deviation in this study. Removing this study from our analysis yielded an indistinguishable overall effect size.

**Fig. 4:**
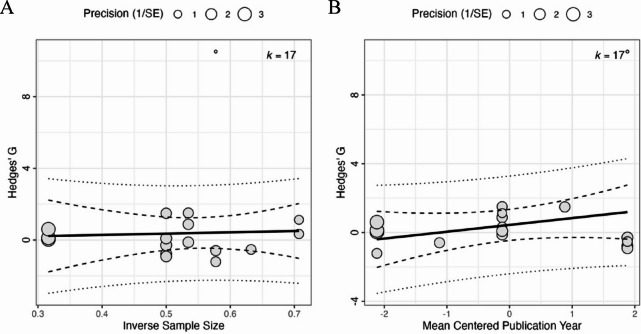
Publication bias for studies on microplastics effect on gut alpha diversity. (**A**) Publication bias against small sample size. (**B**) Publication bias against time delay. The bold line indicates the moderator regression line. The dashed lines indicate the 95% CI and the dotted lines indicate the predictability intervals. *K* denotes the total number of effect sizes in the moderator analysis. Bubble size reflects the weight of the effect size in the meta-analysis. Gut bacteria alpha diversity is estimated by Shannon diversity index.

We found no evidence for small sample size publication bias (0.721, 95% CI − 7.036 to 8.478, z-value = 0.182, *p* = 0.856, Fig. 4A), or time delay publication bias (0.397, 95% CI − 0.254 to 1.048, z-value = 1.194, *p* = 0.232, Fig. 4B).

### Effect of microplastics on the relative abundance of gut bacteria across phyla

**Fig. 5:**
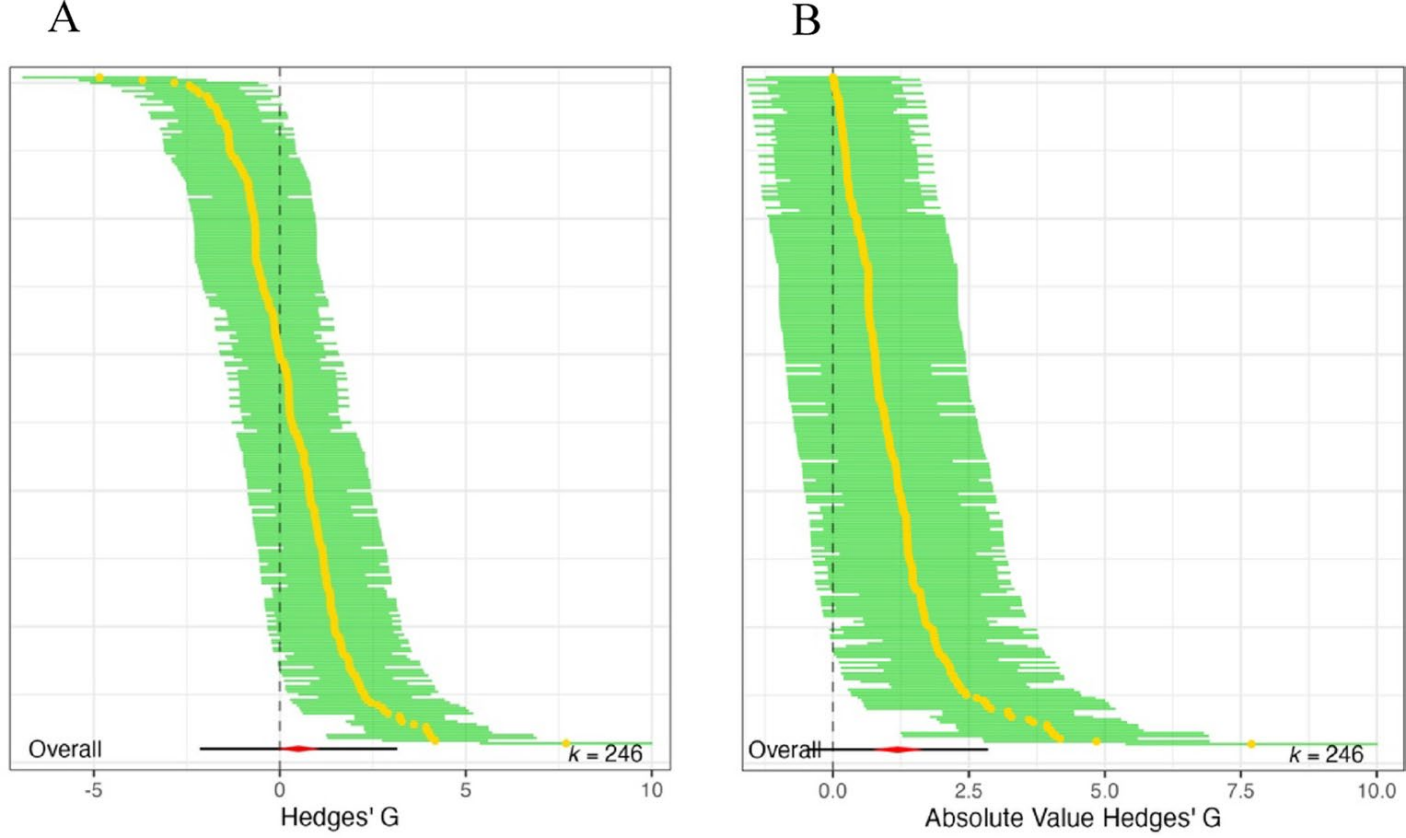
Forest plots of Hedges’ g effect sizes of microplastics impact on gut bacteria relative abundance. (**A**) Relative abundance across phyla. (**B**) Absolute changes in relative abundance across phyla, using the same data. Yellow circles show the meta-analysis effect size point estimate for each set of control vs. treatment means, i.e. positive effect sizes indicate microplastic treatment increased the relative abundance of gut bacteria across phyla. Horizontal bars indicate the predictability interval. The red diamond indicates the overall effect size estimate where diamond width reflects the 95% confidence interval. *K* denotes the total effect size count.

We found significant heterogeneity in the effect sizes for relative abundance of gut bacteria across phyla (Q = 714.059, *p* < 0.001), but the overall effect size was close to zero and not significant (0.503, 95% CI − 0.082 to 1.089, z-value = 1.685, *p* = 0.092, Fig. 5A). However, as the effects of microplastics could either increase or decrease the relative abundance of specific bacterial species, we also looked at the absolute change in relative abundance in response to the treatment using the same data. We then found a significant overall effect size of 1.189 (95% CI 0.704 to 1.674, z-value = 4.805, *p* < 0.001) suggesting strong evidence for microplastic exposure to induce shifts in the relative abundance of gut bacteria across phyla (Fig. 5B). The negative and positive responses to microplastics treatment shown in Fig. 5A appear to cancel out each other resulting in an overall effect size close to zero. Based on this analysis alone, one could erroneously conclude that microplastics treatment does not affect the gut microbiome composition. Only after looking at the absolute response in Fig. 5B, it became clear that there is strong support for microplastics treatment to affect the gut microbiome composition as the relative abundance of bacteria species appears moderately to strongly affected (either negatively or positively) in many cases.

**Fig. 6:**
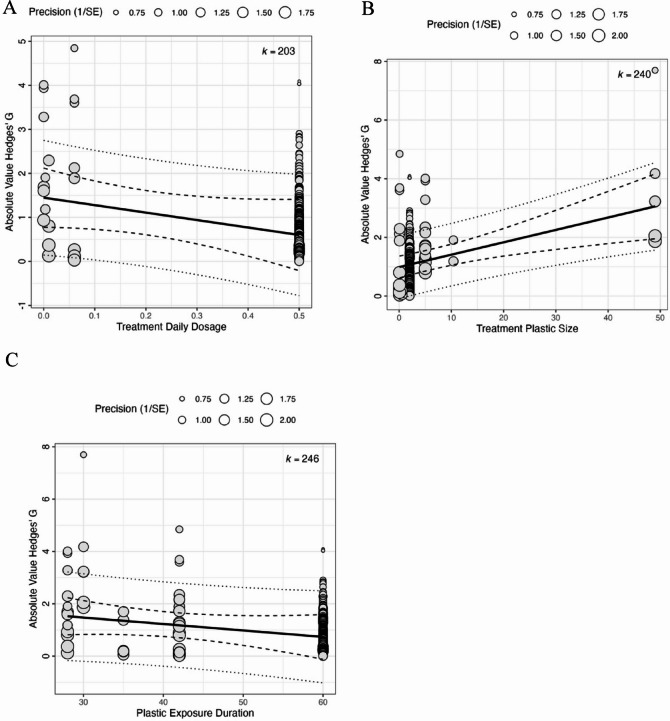
Moderator estimates in the meta-analysis of microplastics impact on relative abundance of gut bacteria. (**A**) Effect of plastic particles daily dosage on gut bacteria relative abundance across phyla. (**B**) Effect of particle size on gut bacteria relative abundance across phyla. (**C**) Effect of plastic exposure duration on gut bacteria relative abundance across phyla. The bold lines indicate the moderators’ regression lines. The dashed lines indicate the 95% CI and the dotted lines indicate the predictability intervals. *K* denotes the total number of effect sizes in the moderator analyses. Bubble size reflects the weight of the effect size in the meta-analysis.

Continuing with the absolute changes in abundance, our moderator analysis revealed the daily dosage of plastic particles in the treatment group was not significant (-1.695, 95% CI − 3.840 to 0.449, z-value = − 1.549, *p* = 0.121, Fig. 6A) nor was the duration of plastic exposure (-0.025, 95% CI − 0.063 to 0.014, z-value = − 1.264, *p* = 0.206, Fig. 6C). On the other hand, the size of plastic particles in the treatment group significantly predicted the absolute response to microplastics exposure (0.042, 95% CI 0.017 to 0.067, z-value = 3.327, *p* < 0.001, Fig. 6B). This finding shows that larger microplastic particles have stronger effects on the relative abundance of gut bacteria across phyla, contrary to what we predicted. While adding these moderators reduced heterogeneity, the residual heterogeneity remained substantial (QE = 165.040, *p* = 0.962).

**Fig. 7:**
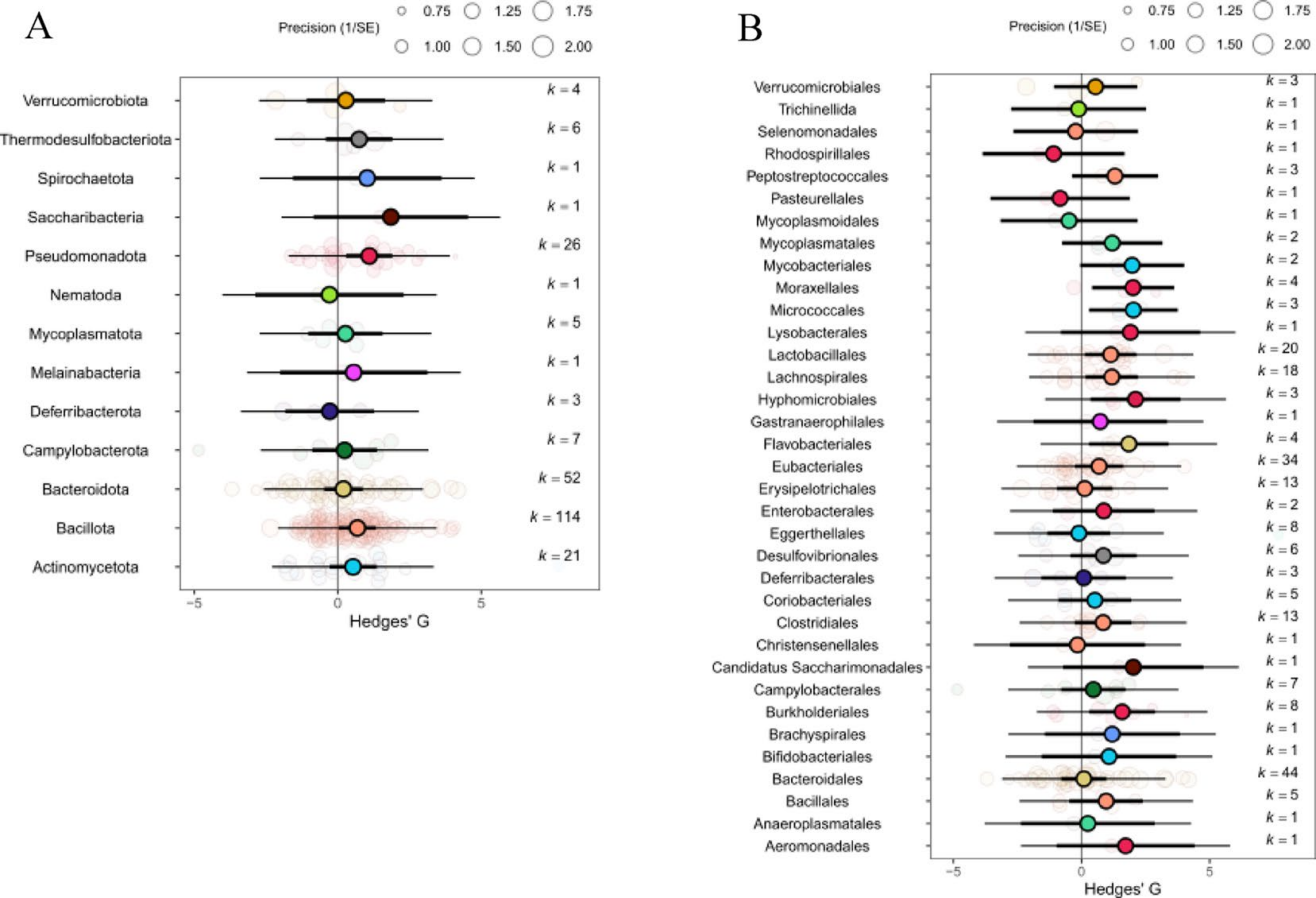
Taxonomic moderator estimates in the meta-analysis of microplastics impact on relative abundance of gut bacteria. (**A**) Effect of microplastic exposure on the relative abundance for each gut bacteria phylum. (**B**) Effect of microplastic exposure on the relative abundance for each gut bacteria order. The bold lines indicate the 95% CI. *K* denotes the total number of effect sizes in the moderator analyses. Bubble size reflects the weight of the effect size in the meta-analysis. Colours indicate corresponding orders per phyla.

Returning to relative changes in abundance, the phyla of bacterial species present in the mice gut microbiome was a significant moderator, reducing heterogeneity compared to the intercept only model, though the residual heterogeneity remained substantial (QE = 687.209, *p* < 0.001). Looking more in detail, the microplastics exposure significantly increased relative abundance for two of the 13 phyla: Bacillota (formerly Firmicutes) (0.684, 95% CI 0.036 to 1.333, z-value = 2.068, *p* = 0.039, Fig. 7A) and Pseudomonadota (formerly Proteobacteria) (1.099, 95% CI 0.292 to 1.906, z-value = 2.668, *p* = 0.008, Fig. 7A).

We further analysed the data at the order level, a finer taxonomic resolution. Order was a significant moderator and reduced heterogeneity compared with the intercept-only model, yet residual heterogeneity remained substantial (Q_E = 592.717, *p* < 0.001). Specifically, microplastic exposure significantly increased relative abundance for 7 of the 35 orders: Burkholderiales (1.586, 95% CI 0.299 to 2.873, z-value = 2.416, *p* = 0.016), Flavobacteriales (1.844, 95% CI 0.291 to 3.398, z-value = 2.328, *p* = 0.020), Hyphomicrobiales (2.108, 95% CI 0.356 to 3.859, z-value = 2.359, *p* = 0.018), Lachnospirales (1.182, 95% CI 0.165 to 2.200, z-value = 2.277, *p* = 0.023), Lactobacillales (1.138, 95% CI 0.135 to 2.141, z-value = 2.223, *p* = 0.026), Micrococcales (2.024, 95% CI 0.293 to 3.755, z-value = 2.292, *p* = 0.022), and Moraxellales (2.009, 95% CI 0.410 to 3.609, z-value = 2.462, *p* = 0.014), Fig. 7B. This highlights the need to consider the possibility of taxa specific responses.

**Fig. 8:**
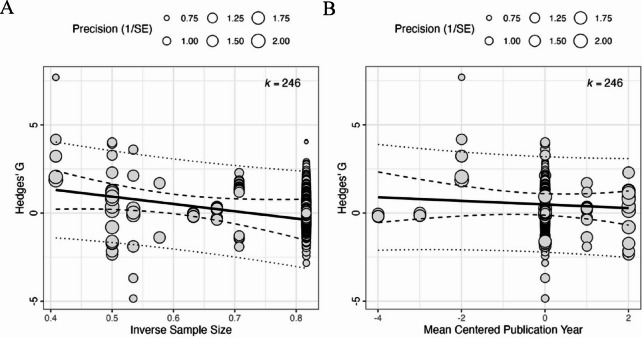
Publication bias for studies on microplastics effect on relative abundance of gut bacteria across phyla. (**A**) Publication bias against small sample size. (**B**) Publication bias against time delay. The bold line indicates the moderator regression trend line. The dashed lines indicate the 95% CI and the dotted lines indicate the predictability intervals. *K* denotes the total number of effect sizes in the moderator analysis. Bubble size reflects the weight of the effect size in the meta-analysis.

Finally, we found no evidence for small sample size publication bias (− 4.142, 95% CI − 8.877 to 0.593, z-value = − 1.715, *p* = 0.086, Fig. 8A), or time delay publication bias (− 0.102, 95% CI − 0.440 to 0.237, z-value = − 0.589, *p* = 0.556, Fig. 8B).

## Discussion

This is the first meta-analysis that tested if experimental dietary exposure to microplastics affects the gut microbiome. Contrary to our initial hypothesis, we did not find a significant overall impact of microplastic exposure on relative abundance of gut bacteria across phyla and Shannon diversity index. However, as microplastics could induce dysbiosis which is typically manifested in a disrupted microbiome balance, the relative abundance of specific species could both decrease or increase. We therefore looked at the absolute change in response to dietary microplastics exposure and then found a significant overall impact of exposure on relative abundance, indicative of heterogeneous effects of microplastics on specific species across phyla. More in-depth meta-analysis revealed two of the 13 bacteria phyla that were investigated (Bacillota and Pseudomonadota) showed a significant positive response to microplastics exposure (Fig. 7A). These phyla-specific effects indicate that certain bacterial phyla in the gut microbiome may be relatively more resilient to the influences of the accumulation of microplastics in the gut epithelia, possibly enabling them to thrive and outcompete more vulnerable phyla^8,27,28^. Improved phyla level differentiation in future studies will be crucial for improving our understanding of the health consequences of microplastics ingestions as different types of bacteria may have differing functions in the gut microbiome. Our current meta-analysis may therefore not provide the full picture about the detailed interplay between microplastics, microbiome dynamics, and their health implications. Our finding of a significant increase in two specific phyla raises important questions, which we examine in the next paragraphs.

*How comprehensive are the 13 phyla currently included in our analysis, and could there be other important phyla that have not yet been considered?*

The 13 included phyla in our meta-analysis represent extractable data from 19 studies. Characterization of the gut microbiome of n = 600 lab mice has revealed at least 29 classifiable phyla^38^. The most abundant phyla found in this study were Bacillota at 55.75%, Bacteroidota at 37.02%, Pseudomonadota at 4.05%, Actinomycetota at 1.98%, and Tenericutes (also known as Mycoplasmatota) at 1.09%, with all other phyla at less than 1%^38^. All five of these were reflected in our 13 phyla from extracted data, which would indicate that despite a total of at least 29 classifiable phyla found in lab mice, our picture of 13 phyla is relatively comprehensive as we have already included eight phyla that make up less than 1% of the microbiome of lab mice. Nonetheless, it is important to note that natural mice populations typically have more diverse microbiome compositions compared to lab mice, which represents a limitation of studies that use lab mice^39^.

*Does increased relative abundance of Bacillota and Pseudomonadota underpin gut dysbiosis?*

Out of the 13 included phyla, the statistically significant increase in the relative abundance of Bacillota and Pseudomonadota was found to be consistent with existing literature. Specifically, a systematic review found most data show microplastic ingestion is associated with dysbiosis where both Bacillota and Pseudomonadota increases, however, looking into Pseudomonadota further, the subgroups of Alphaproteobacteria, Betaproteobacteria and Gammaproteobacteria have been found to decrease^11^*.* This difference between Pseudomonadota increasing as a phylum, but certain subgroups within it decreasing, points to the fact that while phyla-level differentiation is more informative than a blanket diversity index, a phyla is still a broad taxonomic category and further differentiating by more precise taxonomic categories might be needed to derive full understanding of how the microbiome is reacting to microplastic exposure. For example, our order level analysis revealed that out of the three orders within Pseudomonadota showing increased abundance, one belongs to Alphaproteobacteria (i.e., Hyphomicrobiales), one belongs to Betaproteobacteria (i.e., Burkholderiales), and one belongs to Gammaproteobacteria (i.e., Moraxellales). In contrast to our findings of three orders within Pseudomonadota increasing abundance, given that prior literature has found the three Pseudomonadota sub-groups to decrease^11^, this likely points to directional variation of microplastic impact on relative abundance in orders as well. Deeper investigation into the functions of the two phyla that significantly increased in relative abundance tells us Bacillota is important for carbohydrate metabolism in the gut microbiome of humans and is often most dominant by composition in humans and mice, whereas Pseudomonadota has been found to include facultative anaerobes which are often opportunistic pathogens^40^. Thus, while statistically significant changes in these two phyla are indeed indicative of dysbiosis, the significant increase in Pseudomonadota and orders within it may have specific negative implications for healthy microbiome functioning.

*Do specific bacteria species drive the increased relative abundance of Bacillota* and *Pseudomonadota?*

While there is some variation in specific families, genera, or species reported, the common patterns found across studies include an increase in species from the family Muribaculaceae, genus *Lactobacillus*, and genus *Ruminiclostridium* in response to microplastic exposure in mice^41–43^. These patterns are consistent with our analysis as Muribaculaceae is from the phyla Bacteroidota and order Bactereoidales which both showed a non-significant increase in relative abundance in our meta-analysis, *Lactobacillus* is from the phyla Bacillota and order Lactobacillales which both showed a significant increase in relative abundance, and *Ruminiclostridium* is from the phyla Bacillota and order Eubacteriales*,* which showed a significant and non-significant increase, respectively, in relative abundance. Muribaculaceae is associated with regulation of gut and immune function^44^, *Lactobacillus* is associated with carbohydrate metabolism and pathogen protection^45^, and *Ruminiclostridium* is associated with cellulolytic activity and obesity regulation^46^.

*Can microplastic-induced changes in the microbiome be linked to medical conditions such as chronic inflammation?*

Increases in Pseudomonadota are linked with elevated oxidative stress in the intestinal epithelial cells, associated with inflammation^3^. It has been suggested that its increase in relative abundance could reflect a competitive advantage as bacteria in this phyla are relatively aerotolerant and therefore more resistant against oxidative stress^27^. Within Pseudomonadota, the family Enterobacteriaceae has been shown to quickly increase in relative abundance with oxidative stress and inflammation^47^. Whether such changes are symptomatic, or possibly functionally linked with gut health remains unclear. Future studies can incorporate germ free mice to allow for more meaningful studies of causation vs. correlation with inflammation^27^ as well as focus on comparing patterns of relative abundance per bacterial species associated with inflammation such as Enterobacteriaceae*.*

*To what extent are experimental lab manipulations of microplastics realistic and representable for organisms in the natural environment?*

Our meta-analysis supports the existence of experimental effects of microplastics ingestions on the gut microbiome, in controlled laboratory conditions. Whether our findings are relevant for wild animal populations remains to be seen. A limitation of previous captive experimental studies is that they typically use one polymer, consistent with the studies in this meta-analysis where the majority used polystyrene only (Table S1), whereas in the wild, it is likely that animals are exposed to a cocktail of different types of polymers such as polyethylene, polypropylene, and polystyrene^6,8,16^. Captive studies usually use sterile particles, whereas in the wild, microplastics can occur as biofilm^8^ or be coated with pesticides and other toxic chemicals^43^. For these reasons, previous laboratory studies might underestimate the true health impacts of microplastics in natural populations. On the other hand, the exposure to microplastics in natural populations is likely to be much more variable compared to these controlled laboratory experiments. In this context, our finding that dosage does not significantly affect the microbiome is of interest. It could be that gut dysbiosis might only occur after a certain threshold of microplastics exposure has been reached. Under this scenario, the magnitude of health impacts may not be linearly related to the doses of microplastics exposure. In addition to dosage, particle size is likely to be important. We found stronger effects from larger particle sizes (Fig. 6B). This was unexpected, given previous studies found smaller particles can more easily penetrate into the gut epithelial tissues causing tissue damage^48^. It could be possible that larger particles are more likely to physically abrade tissues while they are traveling through the host, harming the digestive tract, contributing to dysbiosis^49^. Finally, as microplastics in the natural environment are likely to occur as cocktails of varying types and sizes, it remains unclear whether these different attributes could interact in a multiplicative manner, with each other, and/or with other environmental pollutants, exacerbating their impacts on health. To begin addressing such questions we need experimental studies on outbred, or non-model organisms using realistic doses and composition of microplastics. For example, a recent study investigated the effects of naturally occurring doses of microplastics on growth, oxidative stress and gut telomere length in African clawed frogs and this study found no significant treatment effects on oxidative stress and gut epithelial telomere length^50^. More studies like these will be essential for improving our understanding of the health consequences of environmental microplastics pollution. Finally, it will be particularly important to increase our understanding of such effects in terrestrial populations as the current focus has largely been on the impacts of microplastics on species in marine environments^2,8,13^.

## Conclusions

Given existing studies on the effects of microplastic exposure on the microbiome demonstrated contrasting findings, this meta-analysis was performed to test if experimental dietary exposure to microplastics affects the gut microbiome. Our results demonstrated support for a significant impact of microplastic exposure on the relative abundance of gut bacteria, and specifically, for an increase in the relative abundance of certain gut bacterial phyla such as Bacillota and Pseudomonadota, and orders within them*.* Deeper analysis into these phyla tell us that subgroups within Pseudomonadota*,* for example, have shown contrasting directional impacts from microplastics^11^, emphasizing future studies may further differentiate by more precise taxonomic categories beyond phyla such as species for more discrete patterns of impact to be studies. Future studies may also include non-model organisms with a mix of plastic polymer types for greater translation to wild populations^6,8,16^, germ free mice for more meaningful differentiation of causation vs. correlation with inflammation^27^, or more terrestrial organisms as they are relatively understudied today.

## Supplementary Information

Below is the link to the electronic supplementary material.

Additional file 1 Table S1.xlsx includes Table S1. Studies Included in this meta-analysis (n = 19). Table S1 lists out the treatment daily dosage (mg/day), average treatment plastic size (μm), plastic exposure duration (days), plastic type, and available phyla for the 19 studies whose data was included in this meta-analysis.

Additional file 2 Shannon index data.csv includes the raw data for the first meta-analysis on gut bacteria species alpha diversity as measured by Shannon index.

Additional file 3 Shannon index meta-analysis.Rmd includes the R scripts for the first meta-analysis on gut bacteria species alpha diversity as measured by Shannon index.

Additional file 4 Relative abundance data.csv includes the raw data for the second meta-analysis on the relative abundance of gut bacteria across phyla.

Additional file 5 Relative abundance meta-analysis.Rmd includes the R scripts for the second meta-analysis on the relative abundance of gut bacteria across phyla.

## Data Availability

All data generated or analyzed during this study are included in this published article (and its supplementary information files).
